# The C-terminus region of β-arrestin1 modulates VE-cadherin expression and endothelial cell permeability

**DOI:** 10.1186/1478-811X-11-37

**Published:** 2013-05-28

**Authors:** Jagoda K Hebda, Héloïse M Leclair, Sandy Azzi, Célestin Roussel, Mark GH Scott, Nicolas Bidère, Julie Gavard

**Affiliations:** 1 Cnrs, UMR810, Institut Cochin, 22 rue Mechain, Rm. 306, Paris, 75014, France; 2 Inserm, U1016, 22 rue Mechain, Paris, 75014, France; 3 Universite Paris Descartes, Sorbonne Paris Cite, 6 rue de l’Ecole de Medecine, Paris, 75006, France; 4 Mitologics, Hopital Robert Debre, 48 Boulevard Serurier, Paris, 75019, France; 5 INSERM UMR_S1014, Hopital Paul Brousse, Villejuif, 94800, France; 6 Universite Paris-Sud P11, Orsay, 91400, France; 7 Equipe Labellisée Ligue contre le Cancer, Villejuif, 94800, France

**Keywords:** VE-cadherin promoter, Endocytosis, β-arrestin, VEGF, Permeability

## Abstract

**Background:**

The endothelial specific cell-cell adhesion molecule, VE-cadherin, modulates barrier function and vascular homeostasis. In this context, we have previously characterized that VEGF (vascular endothelial growth factor) leads to VE-cadherin phosphorylation, β-arrestin2 recruitment and VE-cadherin internalization in mouse endothelial cells. However, exactly how this VE-cadherin/β-arrestin complex contributes to VEGF-mediated permeability in human endothelial cells remains unclear. In this study, we investigated in-depth the VE-cadherin/β-arrestin interactions in human endothelial cells exposed to VEGF.

**Findings:**

First, we demonstrated that VEGF induces VE-cadherin internalization in a clathrin-dependent manner in human umbilical vein endothelial cells (HUVEC). In addition to the classical components of endocytic vesicles, β-arrestin1 was recruited and bound to phosphorylated VE-cadherin. Molecular mapping of this interaction uncovered that the C-terminus tail of β-arrestin1, that comprises amino acids 375 to 418, was sufficient to directly interact with the phosphorylated form of VE-cadherin. Interestingly, the expression of the C-terminus tail of β-arrestin1 induced loss of surface exposed-VE-cadherin, promoted monolayer disorganization and enhanced permeability. Finally, this effect relied on decreased VE-cadherin expression at the transcriptional level, through inhibition of its promoter activity.

**Conclusions:**

Altogether, our results demonstrate that β-arrestin1 might play multiple functions collectively contributing to endothelial barrier properties. Indeed, in addition to a direct implication in VE-cadherin endocytosis, β-arrestin1 could also control VE-cadherin transcription and expression. Ultimately, understanding the molecular mechanisms involved in VE-cadherin function might provide therapeutic tools for many human diseases where the vascular barrier is compromised.

## Findings

The endothelial cells that form blood vessels ensure selective exchanges between plasma and irrigated tissues. Macromolecules and cells larger than 3 nm use the paracellular pathway, chiefly orchestrated through cell-cell junctions [1]. Endothelial junction closing and opening maintain vascular integrity and homeostasis, and coordinate vascular permeability. Adjacent endothelial cells are connected through adherens and tight junctions [2]. VE-cadherin (vascular endothelial cadherin) is the adherens junction protein exclusively expressed in vessels [3]. Besides its key role during vascular network formation, VE-cadherin guarantees barrier integrity and modulates endothelial plasticity in adulthood [4-7]. Many angiogenic molecules can destabilize the organization of intercellular junctions, causing endothelial barrier opening [8-13]. We previously described that vascular endothelial growth factor (VEGF)-induced permeability operates through a signaling pathway involving the sequential activation of Src/Vav2/Rac/PAK [9]. This molecular cascade culminates in VE-cadherin phosphorylation on the highly conserved serine 665 (S665) [9]. This S665 phosphorylation marshals VE-cadherin internalization and subsequent interaction with β-arrestin2, a molecule known for its role in endocytosis of activated membrane receptors [9]. VE-cadherin internalization appears to be a key mechanism that controls the overall organization of endothelial monolayers *in vitro* and vascular organization *in vivo*[9,14-19]. However, how the VE-cadherin/β-arrestin complex is internalized into clathrin-coated vesicles to ultimately cause weakening of adherens junctions, loss of barrier integrity and increased vascular permeability is not yet documented. Here we extend our previous findings [9,19] by demonstrating that, in addition to β-arrestin2, phosphorylated VE-cadherin recruits β-arrestin1. We further mapped a region in the β-arrestin1 C-terminus tail comprising the residues 375 to 418 essential for this interaction. In addition, this domain contributes to the loss of barrier integrity through reduction of VE-cadherin promoter activity.

**Figure 1:**
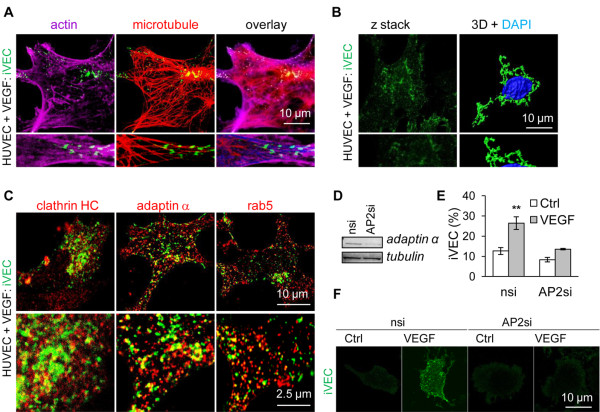
**VE-cadherin is internalized in clathrin coated-vesicles.** (**A**) HUVEC were cultivated on collagen-coated slides, serum deprived, subjected to VE-cadherin internalization assay as described in [18] and stimulated with VEGF (50 ng/ml, 15 min). Cells were fixed and stained for VE-cadherin (iVEC, green), microtubule (red) and actin (purple). Cells were then analyzed by confocal microscopy. Scale bar: 10 μm. (**B**) VE-cadherin staining was performed as described above, analyzed by confocal microscopy and by 3D reconstitution using IMARIS software. Nuclei are presented in blue (DAPI) and iVEC in green. Scale bar: 10 μm. (**C**) Confocal analysis of iVEC (green), together with clathrin heavy chain (HC), adaptin α or rab5 (red) in HUVEC stimulated with VEGF (50 ng/ml, 15 min). Scale bar: 10 μm. (**D-F**) HUVEC received adaptin α-targeting (AP2si) and non-silencing (nsi) duplexes. Total cell lysates were analyzed by western-blot three days later for adaptin α and Tubulin protein expression levels. Confocal analysis of iVEC (green) was performed in the absence (Ctrl) or presence of VEGF (50 ng/ml, 15 min). Graph represents the mean ± s.e.m. of the percentage of cells with iVEC staining; n >300; T-test: ** p < 0.01. Scale bar: 10 μm. All panels are representative of at least 3 independent experiments.

VEGF stimulation leads to VE-cadherin phosphorylation, β-arrestin2 recruitment and VE-cadherin internalization in murine cells [9]. To determine whether human endothelial cells also co-opt this mechanism, we first performed VE-cadherin internalization assays in human umbilical vein endothelial cells (HUVEC) exposed to VEGF (Additional file 1). As early as 15 min, we observed that internalized VE-cadherin vesicles seemed to follow microtubule routes within the cell, in regions where actin filaments were also aligned (Figure 1A). The three-dimensional representation also indicated that VE-cadherin-containing vesicles accumulated in the perinuclear zone, where they could have been transported along microtubules (Figure 1B). VEGF also promoted VE-cadherin internalization in confluent monolayers, although to a lesser extent and with a different pattern (Additional file 2: Figure S1A-B). To further characterize the nature of endocytosed VE-cadherin structures, confocal analysis of internalized VE-cadherin and organelle markers was performed (Figure 1C, Additional file 2: Figure S1C-D). In VEGF-stimulated cells, internalized VE-cadherin co-localized with clathrin-coated vesicles. Accordingly, internalized VE-cadherin co-labeled with adaptin α from the AP2 complex, which is involved in the formation of clathrin-coated pits, and with rab5, a typical marker for early endosomes (Figure 1C). By contrast, no overlap was detected with cholera toxin, which illuminates raft membrane microdomains, caveolin, an essential structural protein for caveolae, or rab11 from the recycling endosomes (Additional file 2: Figure S1C-D). To further investigate the role of the clathrin/AP2-dependent endocytic machinery in this process, endogenous levels of adaptin α were silenced by RNA interference (Figure 1D). In these settings, VEGF-elicited VE-cadherin internalization was highly reduced (Figure 1E-F). Our data clearly establish that short-term VEGF stimulation induces VE-cadherin endocytosis in clathrin-coated vesicles in human endothelial cells.

**Figure 2:**
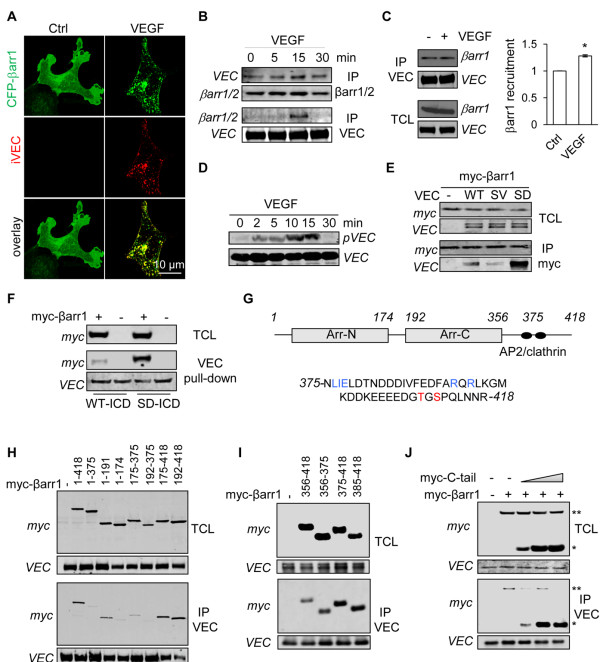
**β-arrestin1 C-terminus tail binds to S665D VE-cadherin mutant.** (**A**) HUVEC were transfected with CFP-myc-tagged β-arrestin1 (CFP-βarr1, green). After serum deprivation, cells were subjected to VE-cadherin internalization assay, stimulated with VEGF (50 ng/ml, 15 min) and fixed for staining (iVEC, red). Cells were analyzed by confocal microscopy. Scale bar: 10 μm. (**B-D**) After overnight serum starvation, three day-old HUVEC were stimulated with VEGF (50 ng/ml) for the indicated times. (**B-C**) The anti-VE-cadherin (VEC) and anti-β-arrestin1/2 (βarr1/2) immunoprecipitated (IP) fractions were analyzed by western-blot. Densitometry analysis was done with Image J software. T-test: * p < 0.05. (**D**) Total cell lysates (TCL) were analyzed by western-blot for S665 phosphorylation of VE-cadherin (pVEC) and total VEC. (**E**) HEK-293T cells were co-transfected with CFP-myc-tagged β-arrestin1 (myc-βarr1) together with control plasmid (−), wild-type (WT), non-phosphorylable (SV) or phosphomimetic (SD) VE-cadherin mutants. TCL and IP fractions were analyzed by western-blot as indicated. (**F**) HEK-293T cells were transfected with CFP-myc-β-arrestin1 (myc-βarr1, +) or CFP-myc control plasmid (−). Cellular extract was incubated with recombinant histidine-fused VE-cadherin intracellular domain (ICD), either WT or SD. TCL and pull-down fractions were analyzed by western-blot as indicated. (**G**) Schematic representation of human β-arrestin1, with two arrestin (arr) domains. The C-terminus tail (375–418) comprises an AP2 binding site (LIE and RXR in blue) and two phosphorylation sites (in red). (**H-J**) HEK-293T cells were co-transfected with VEC SD mutant together with control plasmid (−), full-length and deleted forms of myc-βarr1. IP fractions and TCL were analyzed by western-blot. (**J**) HEK-293T cells were co-transfected with VEC SD mutant together with a constant amount of full-length myc-βarr1 (**) and increasing concentrations of CFP-myc-tagged β-arrestin1 C-terminus tail comprising amino acids 475–418 (myc-C-tail, *). TCL and IP fractions were analyzed by western-blot. All panels are representative of at least 3 independent experiments.

Because β-arrestin1 and β-arrestin2 share redundant functions, we next asked whether β-arrestin1 was similarly involved in VE-cadherin internalization [9,19]. In response to VEGF, β-arrestin1-CFP drastically evolved from a membrane/cytosol diffused localization to a vesicular pattern, which co-localized with VE-cadherin-containing vesicles (Figure 2A, Additional file 2: Figure S1D). Co-immunoprecipitation experiments further showed that VEGF treatment increased a modest but significant VE-cadherin binding to β-arrestin1 (Figure 2B-C). This was accompanied by enhanced VE-cadherin phosphorylation on residue S665 (Figure 2D). We then explored whether VE-cadherin serine phosphorylation impacts on β-arrestin1 recruitment by developing an ectopic expression system in human embryonic kidney cells (HEK-293T). We found that the interaction between β-arrestin1 and a non-phosphorylable mutant (SV) of VE-cadherin was much more limited than with the wild-type (WT) form of VE-cadherin (Figure 2E). By contrast, β-arrestin1 massively bound to a phosphomimetic mutant (SD) of VE-cadherin. Combined, this data strongly suggests that VE-cadherin serine phosphorylation enhances β-arrestin1 recruitment. We next tested the ability of the recombinant WT or SD versions of the intracellular domain of VE-cadherin to pull-down cellular β-arrestin1. Again, SD VE-cadherin strongly bound to β-arrestin1 when compared to WT VE-cadherin (Figure 2F). To further map this association, co-immunoprecipitations were performed in HEK-293T cells co-transfected with SD VE-cadherin and deletion mutants of β-arrestin1 (Figure 2G). While SD VE-cadherin barely bound to β-arrestin1 mutants lacking its C-terminus tail (375–418 amino acids), it efficiently interacted with all deletion mutants that retained this region (Figure 2H). Hence, we defined a 43 amino acid-containing domain required for VE-cadherin/β-arrestin1 interaction. Consistent with this finding, the C-terminus tail of β-arrestin1 was sufficient to interact with SD VE-cadherin (Figure 2I), and efficiently outcompeted full-length β-arrestin1 binding (Figure 2J).

**Figure 3:**
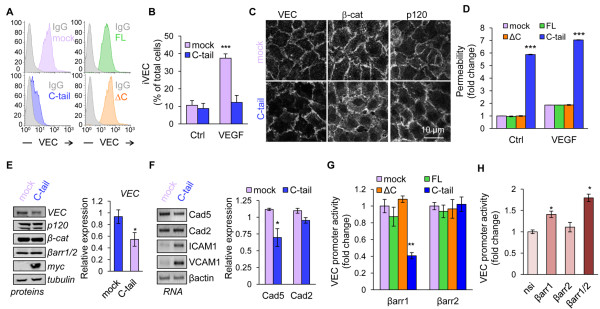
**β-arrestin1 C-terminus tail induces loss of barrier integrity and VE-cadherin down-regulation.** (**A**) VE-cadherin surface expression was tested by flow cytometry in HUVEC that stably expressed mock (pink) or CFP-myc-tagged β-arrestin1 full-length (1–418, green), ΔC (1–374, orange) and C-tail (375–418, blue). Isotype control (Ig) is shown in grey. (**B**) Mock and C-tail HUVEC were subjected to VE-cadherin internalization assay (iVEC) and analyzed by confocal microscopy. Graph represents the mean + s.e.m. of the percentage of cells with iVEC staining; n >300; T-test: *** p < 0.001. (**C**) Three day-old mock and C-tail HUVEC were stained for VE-cadherin (VEC), β-catenin (β-cat) and p120-catenin (p120) and analyzed by confocal microscopy. Scale bar: 10 μm. (**D**) Relative permeability was measured in mock, FL, ΔC and C-tail HUVEC by the fluorescence of 40 kDa FITC-dextran passage. Graph shows the mean ± s.e.m. of 3 independent experiments. Two-way ANOVA test: *** p < 0.001. (**E-F**) Protein and RNA expression of indicated targets were tested using western-blot (E) and RT-PCR (F) in mock and C-tail HUVEC. Cad5: cadherin-5, VE-cadherin; Cad2: cadherin-2, N-cadherin. Densitometry analysis was done with Image J software. T-test: * p < 0.05. (**G-H**) HUVEC were transfected with luciferase reporter for VE-cadherin promoter activity and Renilla. (**G**) They also received a control CFP-myc plasmid (mock) and the CFP-myc-tagged FL, ΔC and C-tail β-arrestin1. Alternatively, they received pGFP (mock) and GFP-tagged β-arrestin2 comprising amino acids 1–410 (FL), 1–359 (ΔC) and 317–410 (C-tail). (**H**) Two days prior plasmid transfection, cells were treated with non-silencing duplexes (nsi) and siRNA targeting βarr1, βarr2, or both (βarr1/2). Graphs show the mean ± s.e.m. of 3 independent experiments. Two-way and one-way ANOVA test: ** p < 0.01; * p < 0.05. All panels are representative of at least 3 independent experiments.

We further investigated the effect of β-arrestin1 C-terminus tail enforced expression in HUVEC. To this end, we engineered HUVEC cells stably expressing β-arrestin1 C-terminus tail (C-tail), along with empty plasmid (mock), full-length (FL) and deleted C-tail (ΔC-tail). The stable expression of CFP-myc-tagged β-arrestin1 constructs was validated by flow cytometry and confocal analysis (Additional file 3: Figure S2A-B). Of note, C-tail preferentially accumulated in the nucleus (Additional file 3: Figure S2B). Flow cytometric analysis further unveiled that surface-exposed VE-cadherin was minimized in C-tail, but not in FL and ΔC-expressing HUVEC (Figure 3A). In agreement with this data, VE-cadherin internalization was not observed in C-tail HUVEC, while VEGF triggered a four-fold increase in VE-cadherin endocytosis in control cells (Figure 3B). These results prompted us to analyze adherens junction organization by confocal microscopy. Corroborating our data, VE-cadherin, p120 and β-catenin appeared less cohesively organized at the cell-cell junctions in C-tail HUVEC, where gaps, zigzags and weaker VE-cadherin staining were observed, resulting in an overall alteration of endothelial monolayer architecture (Figure 3C). Importantly, this impacted on endothelial permeability as measured *in vitro* with a 40 kDa fluorescent tracer (Figure 3D). Indeed, C-tail cells exhibited higher basal endothelial permeability, which could not be further increased by VEGF, when compared to mock, FL and ΔC HUVEC (Figure 3D). To gain insights into the mechanisms involved, we investigated the protein expression levels of adherens junction molecules in C-tail HUVEC. Interestingly, VE-cadherin protein expression was diminished in C-tail cells, while β-catenin, p120-catenin and β-arrestin1/2 remained unchanged (Figure 3E). Following these observations, we examined mRNA levels of VE-cadherin (Cad5), N-cadherin (Cad2) and other endothelial adhesive molecules, such as ICAM1 and VCAM1 (Figure 3F). Our data show that VE-cadherin mRNA was reduced by a quarter in C-tail cells, arguing in favor of a transcriptional effect of the β-arrestin1 C-terminus tail. The lack of change in N-cadherin mRNA suggests that it cannot substitute for VE-cadherin. Interestingly, whereas TNFα challenge strongly increased ICAM1 and VCAM1 expression concomitantly with AP1 and NF-κB activation, neither of these pathways, nor p38 phosphorylation were turned on in C-tail expressing cells (Additional file 4: Figure S3A-D). Finally, the effect of the β-arrestin1 C-terminus tail on the activity of the VE-cadherin promoter [20] was tested in HeLa and HUVEC cells. The β-arrestin1 C-terminus tail expression resulted in a significant decrease in VE-cadherin promoter activity (Figure 3G, Additional file 5: Figure S4A) in both cell lines, although milder in HUVEC. This is most likely due to differences between endothelial and non-endothelial cells [20]. This prompted us to test the possibility that the C-terminus tail of β-arrestin1 binds to the VE-cadherin promoter. Indeed, chromatin immunoprecipitation experiments showed that β-arrestin1 C-tail interacted with the proximal region of the VE-cadherin promoter (Additional file 3: Figure S2C). Interestingly, the expression of the corresponding constructs in β-arrestin2 was unable to alter VE-cadherin promoter activity (Figure 3G, Additional file 5: Figure S4A). This is in agreement with studies showing a predominant role for β-arrestin1 in transcriptional regulation, compared to β-arrestin2, which is actively exported from the nucleus [21,22]. Conversely, reducing β-arrestin1, but not β-arrestin2, expression with siRNA resulted in elevated VE-cadherin promoter activity (Figure 3H, Additional file 5: Figure S4B-C). Hence, the effects of endogenous β-arrestin1 siRNA on VE-cadherin promoter cannot mirror its overexpression. Indeed, β-arrestin1 is expressed in a closed conformation in which the C-terminus tail is masked [23]. Upon activation, conformational changes allow transition to an open state, unleashing the C-tail. This mechanism has been proposed to regulate β-arrestin1 functions [23,24]. In agreement, ectopic C-tail but not full-length β-arrestin1 overexpression curtailed VE-cadherin promoter activity.

Altogether, our data extend our previous findings [9,19] by demonstrating that VEGF induces the recruitment of β-arrestin1 in the course of VE-cadherin internalization in clathrin-containing vesicles. Molecular mapping of VE-cadherin/β-arrestin1 interaction supports the idea of a specific and direct interaction between the C-terminus tail of β-arrestin1 and the phospho-mimicking mutant of VE-cadherin. Additionally, β-arrestin1 C-terminus tail reduces VE-cadherin expression through the inhibition of its promoter activity, suggesting that this multifaceted protein could control endothelial barrier properties by several means. Although our study is mainly based on β-arrestin1 C-terminus tail overexpression, our results might be therapeutically relevant in the context of pathologies exhibiting sustained increase of vascular permeability, especially where endothelial cells are exposed to aberrant levels of VEGF, such as in cancers.

## Abbreviations

CFP: Cyan fluorescent protein; HUVEC: Human umbilical vein endothelial cell; ICAM: Intercellular adhesion molecule; VCAM: Vascular cell adhesion molecule; VE-cadherin: Vascular endothelial cadherin; VEGF: Vascular endothelial growth factor.

## Competing interests

The authors declare no competing interests.

## Authors’ contributions

JKH, HML, SA, CR, NB and JG performed experiments; JKH, HML, SA, NB, and JG analyzed data; MGHS contributed essential reagents; JKH and JG wrote the manuscript. All authors read and approved the final manuscript.

## Supplementary Material

Additional file 1 Methods description. Click here for file

Additional file 2: Figure S1 Characterization of internalized VE-cadherin. (**A**-**C**) HUVEC were cultivated on collagen-coated slides, either sparse or confluent, serum deprived (Ctrl), subjected to VE-cadherin internalization assay and stimulated with VEGF (50 ng/ml, 15 min). Cells were fixed and stained for VE-cadherin (iVEC, gray) and analyzed by confocal microscopy. Scale bar: 10 μm. (**B**) Graph represents the mean ± s.e.m. of the percentage of cells with iVEC staining; n > 200; T-test: ^**^ p < 0.01. (**C**) Confocal analysis of iVEC (green), together with caveolin, cholera toxin (CTX), or rab11 (red). Scale bar: 10 μm. (**D**) Pearson’s coefficients were calculated for each indicated staining with iVEC; n > 10. Panels are representive of 3 independent experiments. Click here for file

Additional file 3: Figure S2 β-arrestin1 expression and localization. (**A**-**B**) HUVEC were transfected with the indicated CFP-fused β-arrestin1 constructs, and analyzed by flow cytometry (A) and confocal microscopy (B). Scale bar: 10 μm. Panels are representive of 3 independent experiments. (**C**) Chromatin immunoprecipitations (ChIP) were performed with anti-GFP and preimmune (lg pAb) antibodies on shear cross-linked chromatin preparation from C-tail HUVEC. Three sets of primers flanking regions -541/-390, -132/+18 and -785/-670 on cadherin-5 (Cad5, VE-cadherin) promoter were used. Additional controls included anti-PoIII IP and premmune (lg mAb) and GADPH primers. Panels are representative of 3 independent experiments. Click here for file

Additional file 4: Figure S3 Status of inflammatory signaling pathways in β-arrestin1 C-tail expressing cells. (**A**) HEK-293 T transfected with luciferase reporter for the indicated promoter activity (NF-_K_B and AP1) and Renilla, together with mock, full lenght (FL) and C-tail β-arrestin1 (C-tail). Alternatively, cells were treated with the TNFα (10 μg/ml, 6 h). Graphs shows the mean + s.e.m. of 3 independent experiments, normalized to Renilla. Two-way ANOVA test: ^***^ p < 0.001. (**B**) HeLa transfected with C-tail β-arrestin1 (green) were stained (red) for p65 and cFos and analyzed by confocal. Alternatively, cells were treated with TNFα for 1 h. (**C**) RT-PCR for ICAM1 and VCAM1 was performed in starved HUVEC (-), treated with TNFα (+, 10 μg/ml,6 h) or expressing C-tail and FL β-arrestin1. GAPDH serves as an internal control. (**D**) HEK-293 T (-) transfected with FL and C-tail β-arrestin1 were analyzed by western-blot for phosphorylated (p) p38. Anisomycin-treated cells (60 μm, 15 min) were used as a positive control. Total ERK2 serves as a loading control. All panels are representive of 3 independent experiments. Click here for file

Additional file 5: Figure S4 Impact of β-arrestin1 and β-arrestin2 on VE-cadherin promoter activity. (**A**-**C**) HeLa were transfected with luciferase reporter for VE-cadherin (VEC) promoter activity and Renilla, together with either, a control CFP-myc plasmid (mock) and CFP-myc-tagged β-arrestin1 (βarr1) comprising amino acids 317-410 (C-tail). (**B**-**C**) Alternatively, HeLa (B) and HUVEC (C) received non-silencing duplexes (nsi) and β-arrestin1 (βarr1), β-arrestin2 (βarr2), and β-arrestin1/2 (βarr1/2)-targeting siRNA. VEC promoter activity was measured in a luciferase-based assay (B), and siRNA efficiency was evaluated by western-blot (C), using anti-βarr1 and anti-βarr2 antibodies. Tubulin serves as a loading control. Graph shows the mean ± s.e.m. of 3 independent experiments. Two-way and one-way ANOVA tests: ^***^p < 0.001; ^**^ p < 0.01;^*^ p < 0.05. Panels are representative of 3 independent experiments. Click here for file
