# Supplementary material for: The C-terminus region of β-arrestin1 modulates VE-cadherin expression and endothelial cell permeability
Source: Cell Commun Signal. 2013 May 28;11:37. doi: 10.1186/1478-811X-11-37 (PMC3669046; doi:10.1186/1478-811X-11-37)
Supplement: Additional file 1 — Methods description. [file 1478-811X-11-37-S1.pdf]

Materials and Methods

Cell culture and transfections.
Human Umbilical Vein Endothelial Cells (HUVEC, clone Ea.hy926, [1]), HeLa (ATCC) and Human Embryonic Kidney (HEK-293T, ATCC) cells were maintained in Dulbecco's modified Eagle's medium (DMEM, Invitrogen) supplemented with 10% fetal bovine serum (FBS) and 1% penicillin/streptomycin. HEK-293T and HeLa were transfected using Turbofect (Fermentas) and HUVEC using the nucleofection kit from Amaxa (Lonza) according to the manufacturers' instructions. To obtain stable cell pools, 2.10^5 HUVEC were seeded in a 6-well plate and transfected in serum-free media 24h later using 2 μg of DNA and 3 μl of Lipofectamine LTX (Invitrogen). Cells were placed in selection medium containing 250 mM geneticin (Invitrogen) for 2 weeks. For AP2 gene silencing, 50 nM of commercially available siRNA against adaptin α (EHU089461, validated Mission siRNA, Sigma) was transfected in HUVEC using RNAi max (Invitrogen) as per the manufacturer's instructions. Previously described Dharmacon siRNA duplexes targeting human β-arrestin1, β-arrestin2 and both were used [2]. Non-silencing (Low GC Duplex, Invitrogen) was used as control.

Reagents and antibodies.
Recombinant human vascular endothelial growth factor A (VEGF) and human TNFα were purchased from R&D; anisomycin was from Sigma. The following antibodies were used: rabbit anti-phospho-S665-VE-cadherin [3]; mouse anti-VE-cadherin (BV6 clone, Millipore); goat anti-VE-cadherin, mouse anti-tubulin, mouse anti-N-cadherin, rabbit anti-Rab5, rabbit anti-Rab11, rabbit anti-caveolin, rabbit anti-cFos, rabbit anti-p65, rabbit anti-ERK2, and rabbit anti-β-arrestin2 (Santa Cruz); rabbit anti-β-arrestin1/2, rabbit anti-phospho-p38, and rabbit anti-clathrin heavy chain (D3C6 clone) (Cell Signaling); mouse anti-myc and rabbit anti-β-catenin (Sigma); rat anti-tubulin (YL1/2 clone), rabbit anti-GFP and rabbit anti-VE-cadherin-FITC (Abcam); mouse anti-p120 and mouse anti-adaptin α (BD Biosciences); and rabbit anti-β-arrestin1 (Epitomics). Alexa568-phalloidin, Alexa594-cholera toxin and all secondary Alexa-conjugated antibodies were from Invitrogen.

DNA constructs.
Different lengths from the coding sequence of human β-arrestin1 were sub-cloned between BamH1 and Not1 in pCEFL-CFP-myc expression vector. Wild type (WT), S665V and S665D mutants of human full-length VE-cadherin were described previously [3], as well as 6*His-tagged intracellular domains of VE-cadherin [3]. pGFP-β-arrestin2 full-length (1-410) and deletion mutants (ΔC-tail 1-359 and C-tail 317-410) were described previously [4].

Flow cytometry.
Cells were incubated for 1h at 4°C with FITC-conjugated mouse anti-VE-cadherin (Abcam) and the respective isotype control immunoglobulin in PBS containing 0.5% BSA. After two washes with ice-cold PBS, cells were analyzed by flow cytometry with a FACScalibur (BD Biosciences). Fluorescence data was acquired for 10000 cells and analyzed using CellQuest software (BD Biosciences). Alternatively, CFP fluorescence was measured in HUVEC.

VE-cadherin internalization and immunofluorescence.
VE-cadherin internalization assays were performed as described previously [5], using anti-VE-cadherin BV6 antibody (1/200 dilution, 1 hour, 4°C) prior stimulation with VEGF (50 ng/ml, 15 min, 37°C). Internalization was measured as the ratio between the number of cells exhibiting more than ten VE-cadherin containing vesicles and the total number of cells.

HUVEC monolayers were grown onto collagen-coated coverslips, fixed (PBS-paraformaldehyde 4%), permeabilized (PBS-Triton 0.5%) and blocked (PBS-BSA 3%). Following incubations with primary and secondary antibodies for 1 hour in PBS-BSA 1.5%, samples were mounted (Fluoromount, Sigma and ProLong, Invitrogen), visualized and analyzed under confocal microscopy (SP5, Leica). Three-dimensional reconstructions were performed using the IMARIS software. Pearson's coefficient was measured using the Image J plugin.

Permeability assays.
Endothelial monolayer permeability was assessed by the passage of FITC-conjugated dextran (0.1 mg/ml, 40 kDa, Invitrogen) as previously described [3]. Briefly, 10^5 HUVEC were plated onto 6.5 mm Transwell Collagen-coated 3 µm pore PTFE membrane inserts (Costar) and left for 3 days to form mature monolayers. After 1 hour of VEGF stimulation including 30 minutes with FITC-dextran, each sample from the bottom chamber was read on a fluorescent plate reader (FUSION, Packard BioScience Company). Values were expressed as the ratio between sample and non-stimulated cells from three independent experiments: * for $p < 0.05$, ** for $p<0.01$ and *** for $p<0.001$ using two-way ANOVA test.

Immunoprecipitations, Histidine pull-downs and western-blots.
Protein lysates were extracted in TNT buffer (Tris-HCl 10 mM pH=7.5, NaCl 150 mM, NP-40 1%, Triton X100 1%, EDTA 2mM, proteases inhibitors (Sigma), NaF 25 mM, and sodium orthovanadate 300 µM) and isolated with centrifugation (10000g, 5 min, 4°C). Post-nuclei soluble supernatants were precleared with 20 µl of protein G-Sepharose (Roche, 4°C, 30 min.), then incubated with 1 µg of indicated antibodies in 30 µl of protein G-Sepharose 5 hours at 4°C. After four washes in lysis buffer, immunoprecipitated fractions were denatured in NUPAGE sample buffer (Invitrogen).
Recombinant 6*His-tagged VE-cadherin intracellular domain bound to nickel beads (Invitrogen) were prepared as described [3] and used to pull-down HEK-293T protein lysates.
Electrophoresis were performed on 4-12% acrylamide gradient gels as per the manufacturer's instructions (NUPAGE, Invitrogen) and transferred onto PVDF membranes (Thermo Scientific) for further analysis using the infrared scanner system (LiCOR).

Luciferase assays.
Firefly luciferase constructs downstream of the VE-cadherin promoter (-3499/-5 region [6]) were co-transfected with renilla luciferase pRL-TK plasmid (Promega) in HUVEC and HeLa, together with 250 ng of the corresponding empty plasmids (pCEFL-myc-CFP and pGFP) alone or in fusion with the β-arrestin1 and β-arrestin2 constructs. For β-arrestin1/2 silencing, cells were transfected two days earlier with siRNA duplexes. Alternatively, firefly luciferase constructs downstream of promoters for NF-κB or activator protein 1 (AP-1) were used in HEK-293T as previously described [7]. Luciferase activity was analyzed using the Dual-Luciferase Kit (Promega) and firefly fluorescence units were normalized to renilla luciferase fluorescence units (BMG microplate reader).

RNA extraction and RT-PCR.
RNA was extracted using the Qiagen RNeasy Mini Kit as per the manufacturer's directions. Equal amounts of RNA were reverse transcribed using the Superscript III RT kit (Invitrogen) and the resulting cDNA was used to amplify human VE-cadherin, N-cadherin, ICAM1 and VCAM1 mRNAs by PCR using the JumpStart REDTaq ReadyMix (Sigma) and the following primer sets for cadherin-5 (5'-CCTACCAGCCCAAAGTGTGT-3' and 3'-GACTTGGCATCCCATTGTCT-5'), cadherin-2 (5'-GACAATGCCCCTCAAGTGTT-3' and

3’-CCATTAAGCCGAGTGATGGT-5’), VCAM1 (5’-TAAAATGCCTGGGGAGATGG-3’ and 3’-GGTGGTGGAAGTCAATGAGA-5’), ICAM1 (5’-GGCTGGAGCTGTTTGAGAACA-3’ and 3’-ACTGTGGGGTTCAACCTCG-5’). Human β-actin and GAPDH were also amplified as control for input (5′-AGCACTGTGTTGGCGTACAG-3′ and 3′-GGACTTCGAGCAAGAGATGG-5′) and (5’-GTGGACCTGACCTGCCGTCT-3’ and 3’-GGAGGAGTGGGTGTCGCTGT-5’), respectively. PCR products were separated by electrophoresis on SYBR green-containing agarose gels (Invitrogen).

Chromatin immunoprecipitation

Chromatin immunoprecipitation assays were performed according to manufacturer’s instructions (EZMagna ChIP, Millipore). Briefly, C-tail cells were treated with 1% formaldehyde (RT, 10 min) and harvested in lysis buffer. Chromatin was then sheared by sonication using Bioruptor Diagenesis machine. Immunoprecipitations were performed at 4°C overnight with anti-GFP antibody or anti-PolII (5 µg Abcam and Millipore, respectively) and rabbit and mouse preimmune serum (2 µg, Santa Cruz). Immunocomplexes were collected with Protein G Magnetic beads, washed and eluted. Proteinase K treatment (10 mg/ml, 65°C, 2 hours) was applied to reverse DNA/protein complexes crosslink. Purified DNA was then used for PCR using the following primer sets on VE-cadherin promoter: -541/-390 5’-ATCTCCTGAAGCCTCCCTGT-3’ and 3’-GGGTTTAAGGTGCTTGTCCA-5’; -785/-670 5’-CCTTGTGAGGATGAGGCTGT-3’ and 3’-GGAGTCAAGTGACCCAGCTC-5’; -132/+18 5’-AGCCAGCCCAGCCCTCACA-3’ and 5’-CCTGTCAGCCGACCGTCTTT-3’. GAPDH primers were from the EZMagna ChIP Millipore kit.

References

1. Edgell CJ, McDonald CC, Graham JB: **Permanent cell line expressing human factor VIII-related antigen established by hybridization**. *Proc Natl Acad Sci U S A* 1983, **80**(12):3734-3737.
2. Molla-Herman A, Boularan C, Ghossoub R, Scott MG, Burtey A, Zarka M, Saunier S, Concordet JP, Marullo S, Benmerah A: **Targeting of beta-arrestin2 to the centrosome and primary cilium: role in cell proliferation control**. *PLoS One* 2008, **3**(11):e3728.
3. Gavard J, Gutkind JS: **VEGF controls endothelial-cell permeability by promoting the beta-arrestin-dependent endocytosis of VE-cadherin**. *Nat Cell Biol* 2006, **8**(11):1223-1234.
4. Scott MG, Le Rouzic E, Perianin A, Pierotti V, Enslen H, Benichou S, Marullo S, Benmerah A: **Differential nucleocytoplasmic shuttling of beta-arrestins. Characterization of a leucine-rich nuclear export signal in beta-arrestin2**. *J Biol Chem* 2002, **277**(40):37693-37701.
5. Xiao K, Allison DF, Buckley KM, Kottke MD, Vincent PA, Faundez V, Kowalczyk AP: **Cellular levels of p120 catenin function as a set point for cadherin expression levels in microvascular endothelial cells**. *J Cell Biol* 2003, **163**(3):535-545.
6. Prandini MH, Dreher I, Bouillot S, Benkerri S, Moll T, Huber P: **The human VE-cadherin promoter is subjected to organ-specific regulation and is activated in tumour angiogenesis**. *Oncogene* 2005, **24**(18):2992-3001.
7. Dwyer J, Hebda JK, Le Guelte A, Galan-Moya EM, Smith SS, Azzi S, Bidere N, Gavard J: **Glioblastoma cell-secreted interleukin-8 induces brain endothelial cell permeability via CXCR2**. *PLoS One* 2012, **7**(9):e45562.
