# Supplementary figures and images for: The C-terminus region of β-arrestin1 modulates VE-cadherin expression and endothelial cell permeability
Source: Cell Commun Signal. 2013 May 28;11:37. doi: 10.1186/1478-811X-11-37 (PMC3669046; doi:10.1186/1478-811X-11-37)

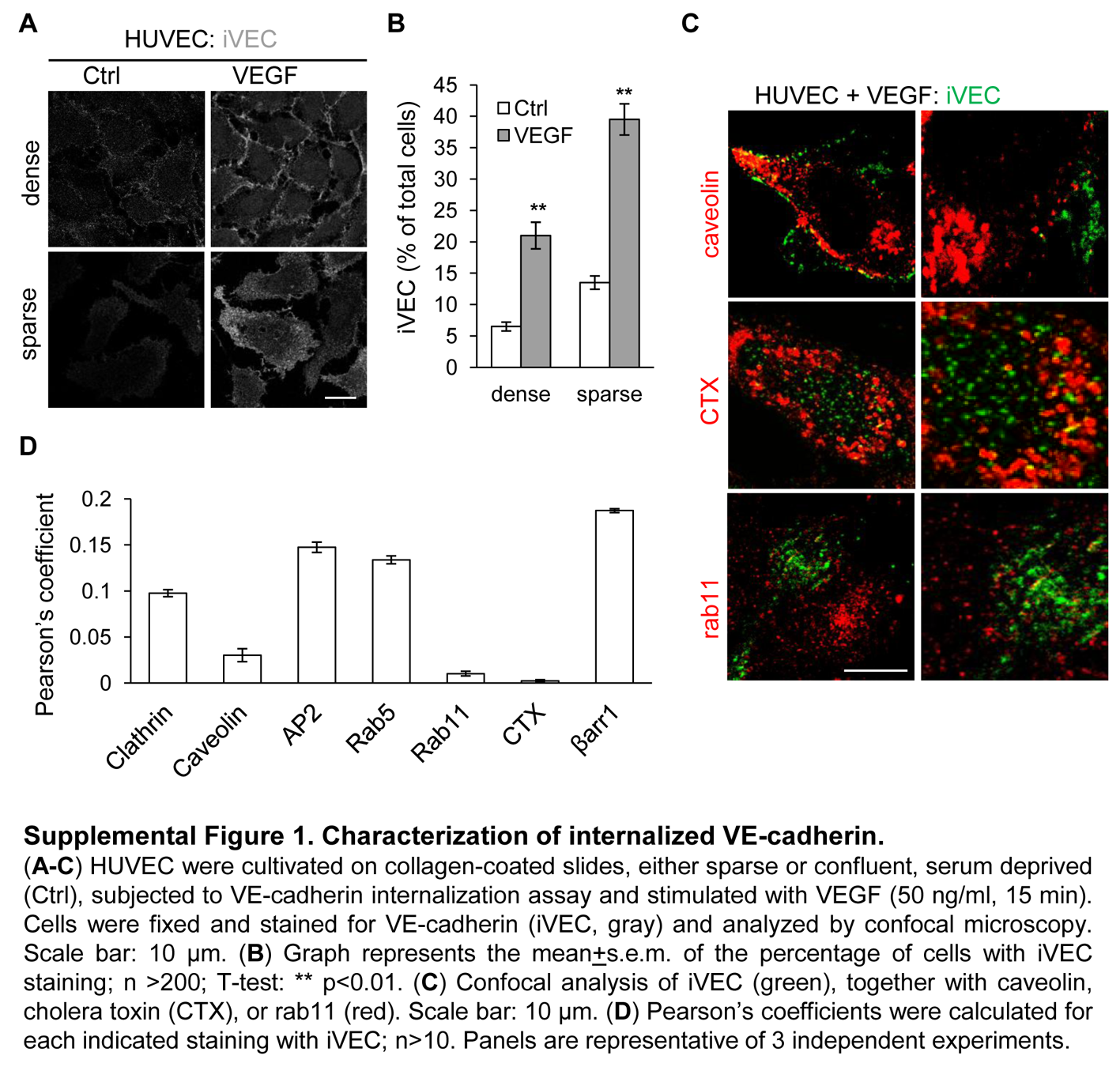

Supplement: Additional file 2: Figure S1 — Characterization of internalized VE-cadherin. (A-C) HUVEC were cultivated on collagen-coated slides, either sparse or confluent, serum deprived (Ctrl), subjected to VE-cadherin internalization assay and stimulated with VEGF (50 ng/ml, 15 min). Cells were fixed and stained for VE-cadherin (iVEC, gray) and analyzed by confocal microscopy. Scale bar: 10 μm. (B) Graph represents the mean ± s.e.m. of the percentage of cells with iVEC staining; n > 200; T-test: ** p < 0.01. (C) Confocal analysis of iVEC (green), together with caveolin, cholera toxin (CTX), or rab11 (red). Scale bar: 10 μm. (D) Pearson’s coefficients were calculated for each indicated staining with iVEC; n > 10. Panels are representive of 3 independent experiments. [file 1478-811X-11-37-S2.tiff]

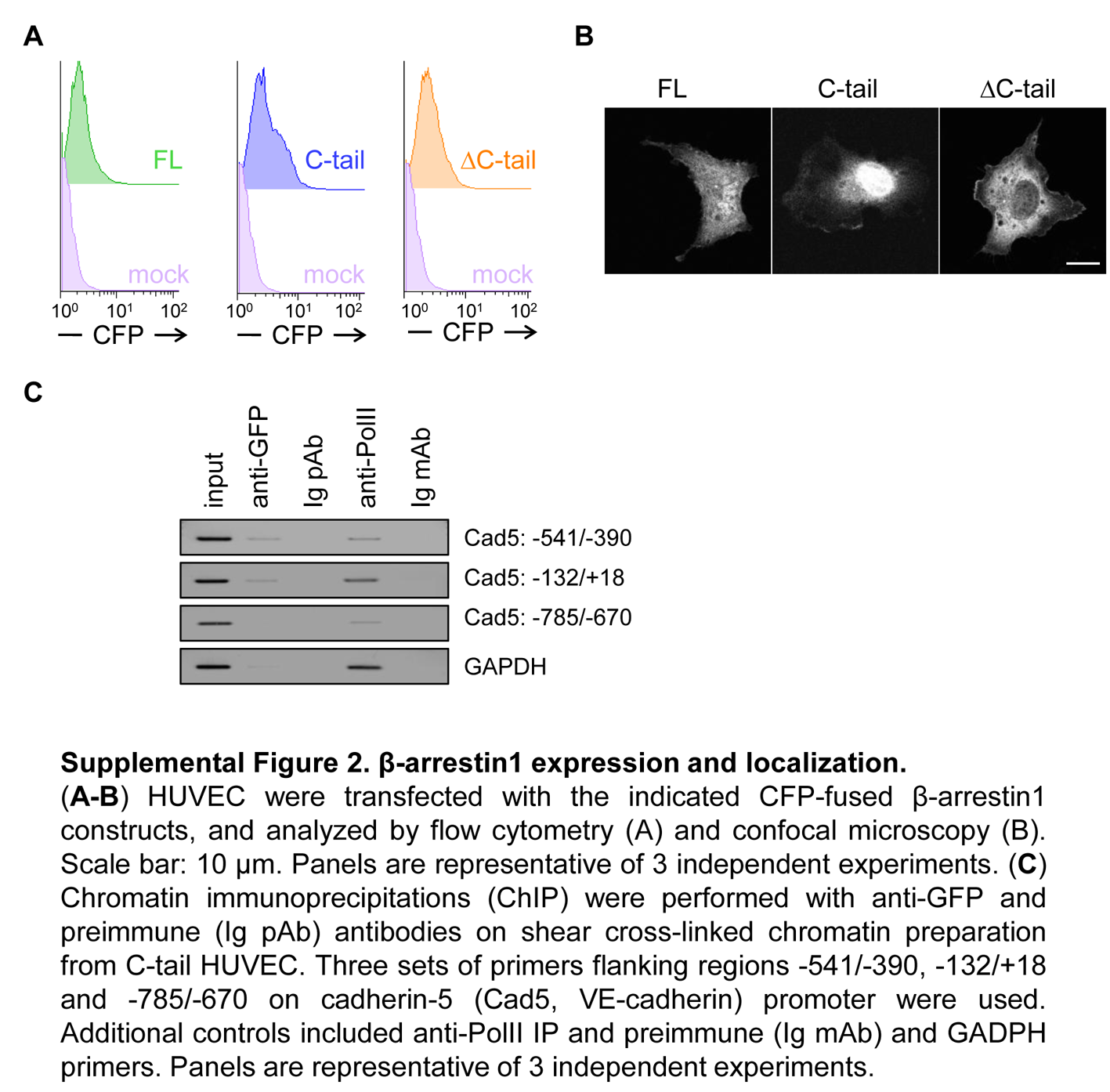

Supplement: Additional file 3: Figure S2 — β-arrestin1 expression and localization. (A-B) HUVEC were transfected with the indicated CFP-fused β-arrestin1 constructs, and analyzed by flow cytometry (A) and confocal microscopy (B). Scale bar: 10 μm. Panels are representive of 3 independent experiments. (C) Chromatin immunoprecipitations (ChIP) were performed with anti-GFP and preimmune (lg pAb) antibodies on shear cross-linked chromatin preparation from C-tail HUVEC. Three sets of primers flanking regions -541/-390, -132/+18 and -785/-670 on cadherin-5 (Cad5, VE-cadherin) promoter were used. Additional controls included anti-PoIII IP and premmune (lg mAb) and GADPH primers. Panels are representative of 3 independent experiments. [file 1478-811X-11-37-S3.tiff]

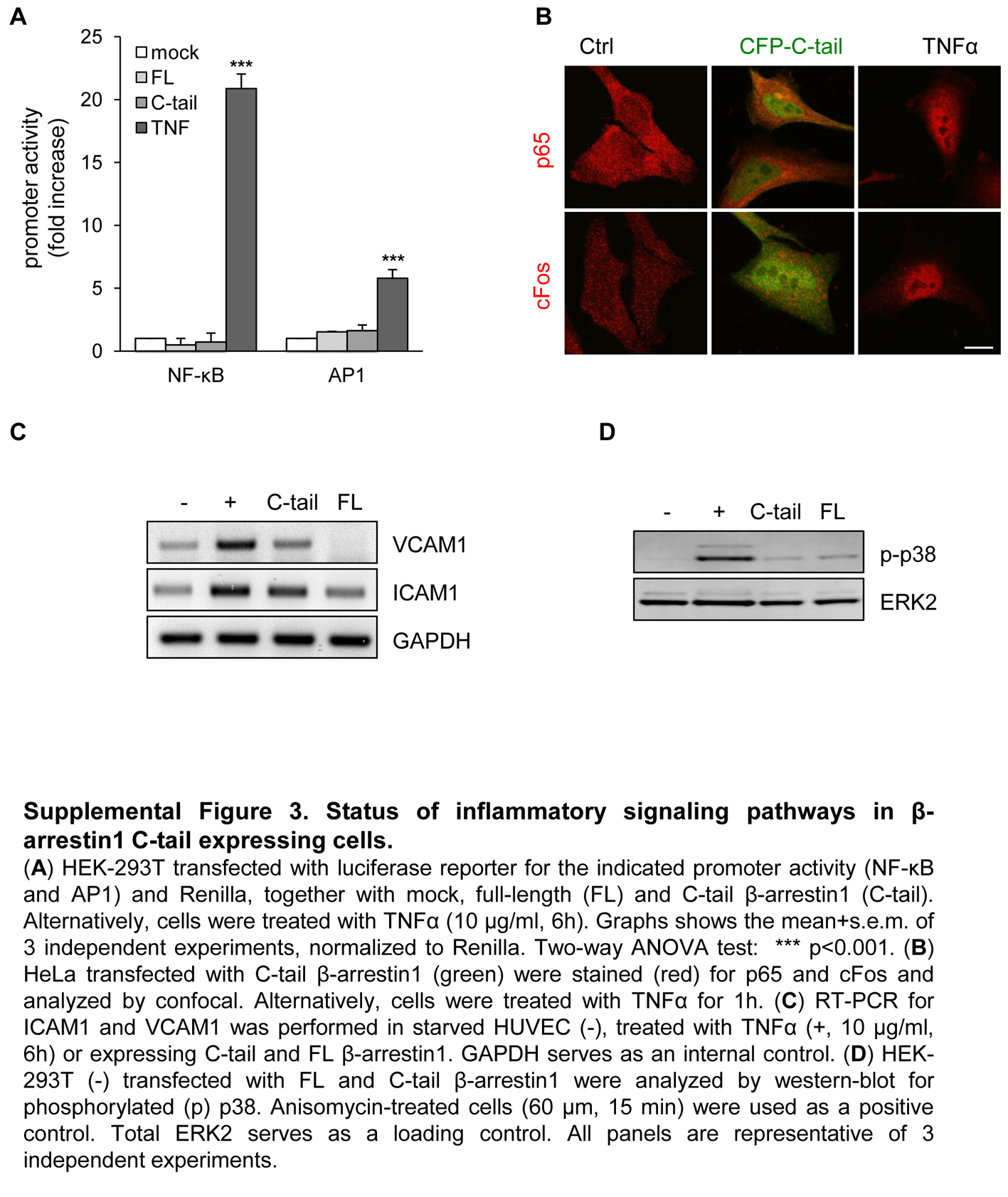

Supplement: Additional file 4: Figure S3 — Status of inflammatory signaling pathways in β-arrestin1 C-tail expressing cells. (A) HEK-293 T transfected with luciferase reporter for the indicated promoter activity (NF-KB and AP1) and Renilla, together with mock, full lenght (FL) and C-tail β-arrestin1 (C-tail). Alternatively, cells were treated with the TNFα (10 μg/ml, 6 h). Graphs shows the mean + s.e.m. of 3 independent experiments, normalized to Renilla. Two-way ANOVA test: *** p < 0.001. (B) HeLa transfected with C-tail β-arrestin1 (green) were stained (red) for p65 and cFos and analyzed by confocal. Alternatively, cells were treated with TNFα for 1 h. (C) RT-PCR for ICAM1 and VCAM1 was performed in starved HUVEC (-), treated with TNFα (+, 10 μg/ml,6 h) or expressing C-tail and FL β-arrestin1. GAPDH serves as an internal control. (D) HEK-293 T (-) transfected with FL and C-tail β-arrestin1 were analyzed by western-blot for phosphorylated (p) p38. Anisomycin-treated cells (60 μm, 15 min) were used as a positive control. Total ERK2 serves as a loading control. All panels are representive of 3 independent experiments. [file 1478-811X-11-37-S4.tiff]

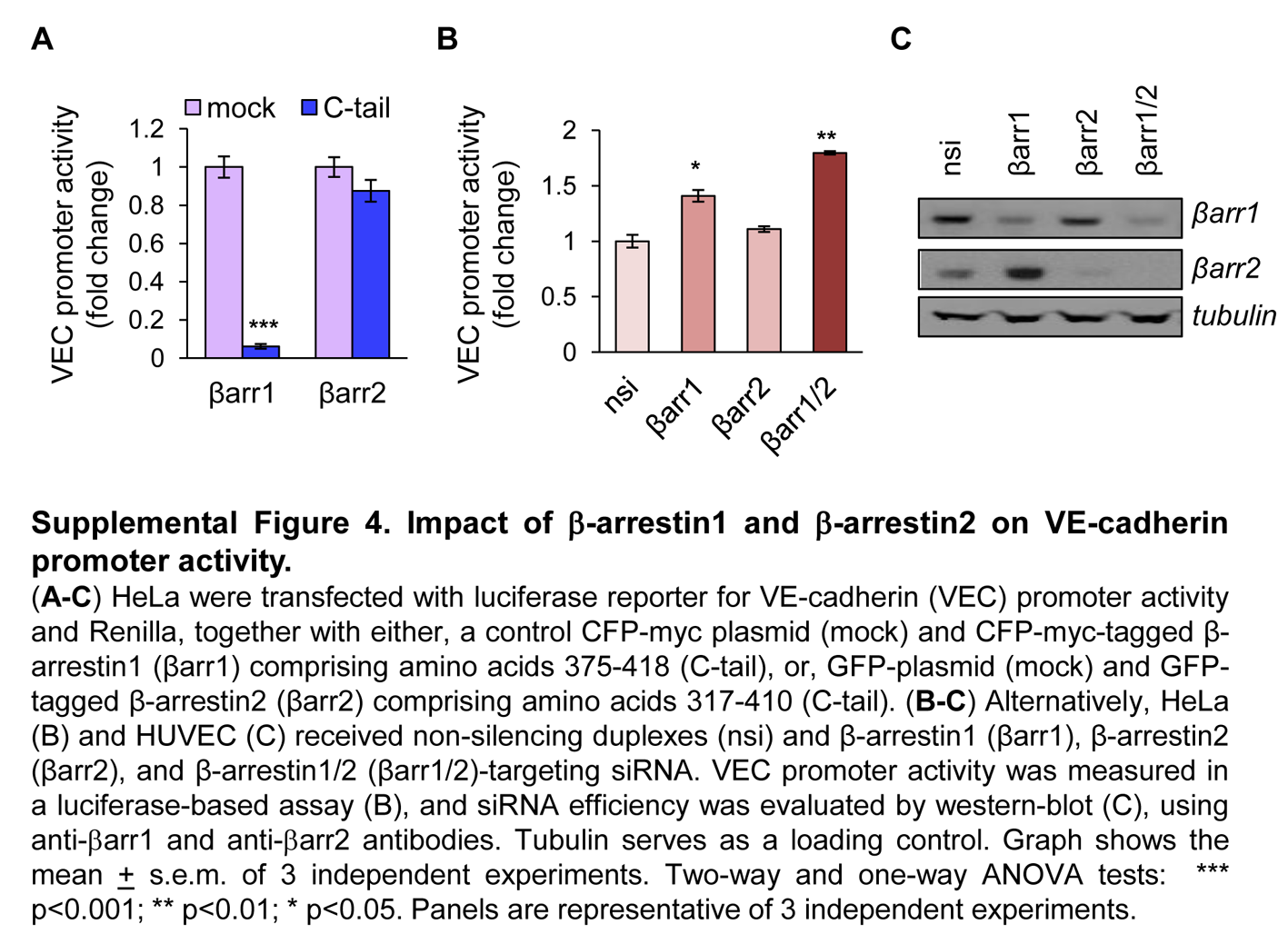

Supplement: Additional file 5: Figure S4 — Impact of β-arrestin1 and β-arrestin2 on VE-cadherin promoter activity. (A-C) HeLa were transfected with luciferase reporter for VE-cadherin (VEC) promoter activity and Renilla, together with either, a control CFP-myc plasmid (mock) and CFP-myc-tagged β-arrestin1 (βarr1) comprising amino acids 317-410 (C-tail). (B-C) Alternatively, HeLa (B) and HUVEC (C) received non-silencing duplexes (nsi) and β-arrestin1 (βarr1), β-arrestin2 (βarr2), and β-arrestin1/2 (βarr1/2)-targeting siRNA. VEC promoter activity was measured in a luciferase-based assay (B), and siRNA efficiency was evaluated by western-blot (C), using anti-βarr1 and anti-βarr2 antibodies. Tubulin serves as a loading control. Graph shows the mean ± s.e.m. of 3 independent experiments. Two-way and one-way ANOVA tests: ***p < 0.001; ** p < 0.01;* p < 0.05. Panels are representative of 3 independent experiments. [file 1478-811X-11-37-S5.tiff]
